# Effect of ankle joint position on triceps surae contractile properties and motor unit discharge rates

**DOI:** 10.14814/phy2.14680

**Published:** 2020-12-23

**Authors:** Kalter Hali, Alexander M. Zero, Charles L. Rice

**Affiliations:** ^1^ Faculty of Health Sciences School of Kinesiology The University of Western Ontario London ON Canada; ^2^ Department of Anatomy and Cell Biology Schulich School of Medicine and Dentistry The University of Western Ontario London ON Canada

**Keywords:** EMG, gastrocnemius, muscle length, soleus

## Abstract

The triceps surae (TS) length–tension relationship can be altered by changing the knee joint position, ankle joint position or both. However, studies exploring the effect of muscle length on neuromuscular properties have focused only on knee joint position changes affecting two of the three muscle components of the TS. Thus, the purpose of this study is to compare the neuromuscular properties of the three TS muscles during plantar flexion contractions at two ankle joint positions, 20° dorsiflexed (DF) and 20° plantar flexed (PF). Maximal isometric voluntary strength (MVC), voluntary activation, and evoked contractile properties of the ankle plantar flexors were compared between both ankle joint positions. Additionally, soleus, medial (MG), and lateral (LG) gastrocnemii motor unit discharge rates (MUDRs) were sampled during plantar flexion contractions at 25%, 50%, 75%, and 100% MVC using indwelling tungsten electrodes. MVC and peak twitch torque were lower by ~61% and 70%, respectively, whereas the maximal rate of torque relaxation was 39% faster in the PF compared with the DF position. Voluntary activation (~95%) was unaffected by changes in ankle joint position. LG MUDRs showed no differences between ankle joint positions, regardless of contraction intensity. Submaximal MG and soleus MUDRs showed no differences between the two ankle joint positions, however both muscles had 9% and 20% higher MUDRs in the DF position, respectively. These results provide further evidence for the differential activation among the three components of the TS with the greatest increases in soleus MUDRs compared with the gastrocnemii when the muscles are lengthened.

## Graphical abstract

Maximal motor unit discharge rates (MUDRs) of the medial gastrocnemius and soleus are lower when these muscles are shortened by changing the ankle joint position, whereas lateral gastrocnemius maximal MUDRs are not.

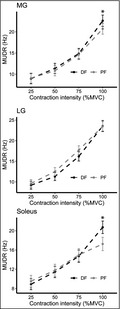

## 1 INTRODUCTION

Studies exploring muscles at different lengths in humans report that when electrically stimulating muscles, higher rates of stimulation are required to reach tetanic fusion in a shortened compared with a lengthened muscle due to faster contractile properties when the muscle is shortened (Gandevia & Mckenzie, 1988; Marsh et al., 1981). Thus, although there are limitations for direct comparisons, in principle, maximal motor unit discharge rates (MUDRs) should be higher in a shortened compared with a lengthened muscle in order for optimal torque fusion to occur. However, only two studies have explored maximal MUDRs across different muscle lengths and report conflicting results. In the tibialis anterior, MUDRs during a maximal voluntary contraction (MVC) showed no difference between muscle lengths (Bigland‐Ritchie et al., 1992). However, in the hamstrings, maximal MUDRs were higher in the shortened compared with the lengthened position (Kirk & Rice, 2017). Furthermore, increases, decreases and no changes have been reported when comparing submaximal MUDRs at different muscle lengths (Bigland‐Ritchie et al., 1992; Christova et al., 1998; Del Valle et al. 2004; Hali et al., 2019; Kennedy & Cresswell, 2001; Kirk & Rice, 2017; Lauber et al., 2014; Pasquet et al., 2005; Vander Linden et al., 1991).

The triceps surae (TS) is a uniquely accessible model to compare MUDRs between different muscle lengths as it is comprised of the bi‐articular medial (MG) and lateral (LG) gastrocnemii together with the mono‐articular soleus, all of which insert into the calcaneal tendon. As such, the length‐tension relationship of the TS can be altered by changes in the angle of the knee joint, ankle joint, or both. Previous work has focused on the effect of changes in the knee joint angle to TS neuromuscular properties. In these studies, the gastrocnemii have demonstrated decreased submaximal MUDRs and increased motor unit recruitment thresholds when placed in a shortened position by flexing the knee joint (Hali et al., 2019; Kennedy & Cresswell, 2001). From surface electromyography (sEMG) recordings, it has been shown that when the knee joint is flexed, there was an increase in soleus activity to compensate for the compromised torque producing capability of the gastrocnemii in this position, despite no length change in the soleus (Kennedy & Cresswell, 2001). However, no changes in soleus MUDRs or motor unit recruitment thresholds were found during plantar flexion contractions in a flexed compared with an extended knee joint position (Hali et al., 2019). Thus, it remains unclear how a change in ankle joint position, which affects the length of all components of the TS (MG, LG, and soleus) affects the neuromuscular properties of this muscle group. Therefore, comparing submaximal and maximal MUDRs in the three components of the TS between two ankle joint positions will provide further insight into how synergistic muscles respond to changes in length during a common task when the muscles are in a shortened compared with a lengthened position.

Furthermore, it has been reported that the components of the TS demonstrate different activation patterns during voluntary plantar flexion contractions with the soleus being recruited first, followed by the MG and then the LG (Hali et al., 2019; Héroux et al., 2014). The soleus is composed of ~85% slow‐twitch muscle fibers, whereas the gastrocnemii are composed of ~50% slow‐twitch muscle fibers (Johnson et al., 1973). Additionally, the soleus has a muscle spindle density ~2.5 fold greater than that of the gastrocnemii (Banks, 2006; Voss, 1971) and receives greater spindle feedback (Tucker & Türker, 2004) making it more likely to be affected by a change in muscle length compared with the gastrocnemii. Therefore, an understanding of how the MUDRs of the TS components are affected by changes in ankle joint position will provide improved insight into how synergistic muscles with different activation patterns and fiber types are affected by muscle length changes during the common task of isometric plantar flexion.

Thus, the purpose of this study is to compare neuromuscular properties of the MG, LG, and soleus at two ankle joint positions, which affect the lengths of all three muscles, during the common task of isometric plantar flexion throughout a range of submaximal and maximal contraction intensities. For this, we recorded maximal voluntary strength, contractile properties, voluntary activation, and submaximal and maximal MUDRs of the TS muscles at two ankle joint positions: 20° dorsiflexed (DF; lengthened) and 20° plantar flexed (PF; shortened). Given the previously reported decrease in MG and LG MUDRs in a shortened position (Hali et al., 2019; Kennedy & Cresswell, 2001), we hypothesized that MG, LG, and soleus MUDRs will be higher in the DF (lengthened) compared with the PF (shortened) position.

## 2 MATERIALS AND METHODS

### 2.1 Participants

Ten males (24 ± 3 years old, 81 ± 7 kg, 181 ± 5 cm) volunteered for the study. The homogenous population in this study was chosen to minimize the influence of sex‐based differences in Achilles tendon stiffness (Intziegianni et al., 2017), which would affect TS muscle length changes with alterations of ankle joint position. All participants were recreationally active, defined as not involved in any varsity level or systematic training activities, but not sedentary, and were considered healthy and free of neuromuscular disease. All participants gave oral and written consent prior to the testing. The study was approved by the local University's Review Board for Health Sciences Research Involving Human Participants (#107505).

### 2.2 Experimental arrangement

Participants were seated upright on a chair with their left leg placed in a custom isometric dynamometer used to record plantar flexion torque (Marsh et al., 1981). Hip and knee joint angles were both at 90°. To avoid any potential training effect from preferential use of the dominant leg during daily activities, the nondominant leg (left) was chosen. The ankle joint angles tested were 20° dorsiflexion (DF; lengthened TS muscles) and 20° plantar flexion (PF; shortened TS muscles) and here we refer to a neutral ankle joint angle as 0°. The foot was secured to the dynamometer using two inelastic straps across the toes and dorsum of the foot and one at the ankle. A metal C‐shaped bar pressing firmly against the distal aspect of the thigh minimized extraneous leg and hip movement during the contractions. Plantar flexion torques were transmitted through a rigid footplate and strain gauge mounted at the joint axis of rotation. Torque was recorded from the dynamometer, analog‐to‐digitally converted (Power 1401; Cambridge Electronic Design), and sampled at 500 Hz (Spike2; Cambridge Electronic Design). Real‐time torque production was displayed on a computer screen ~1 m away from the participant for visual feedback.

All electrically stimulated properties were evoked via stimulation of the tibial nerve at the distal popliteal fossa using a stimulator (Model DS7AH; Digitimer) with a 200 microsecond square wave pulse delivered at 400 V. Current intensity was adjusted until there were no further increases in twitch amplitude, and then increased 20% to ensure supramaximal stimulation (60–120 mA).

### 2.3 Electromyography

Surface EMG from the TS and tibialis anterior were recorded through self‐adhering (GE Healthcare, ECG electrodes) electrodes arranged in a monopolar setup. For a global TS sEMG measure, the active electrode was placed on the border separating the MG, LG, and soleus with the reference electrode placed over the calcaneal tendon. For antagonist coactivation measures, the active electrode was placed over the muscle belly of the tibialis anterior and the reference electrode over its tendon at the ankle. All sEMG signals were preamplified (100×), filtered between 10 Hz and 10 kHz (Neurolog, NL844; Digitimer), and sampled at 2 kHz (Spike2; Cambridge Electronic Design).

Intramuscular EMG recordings were obtained with custom‐made insulated tungsten microelectrodes (123 μm in diameter and 45 mm length; Frederick Haer Company). The insertion sites were cleansed with 70% isopropyl‐alcohol by swabbing the skin surface over the muscle bellies. Two sterile microelectrodes (connected to separate channels) were individually inserted by two operators. The microelectrode EMG signals were preamplified (100×), filtered between 10 Hz and 10 kHz (Neurolog; NL844; Digitimer) and each channel sampled at 20 kHz (Spike2; Cambridge Electronic Design). Reference surface electrodes for the tungsten intramuscular electrodes were placed on the medial and lateral malleoli. A common ground electrode for both sEMG and intramuscular EMG was positioned over the patella. Audio and visual feedback was provided to each operator independently.

### 2.4 Experimental procedure

**FIGURE 1:**
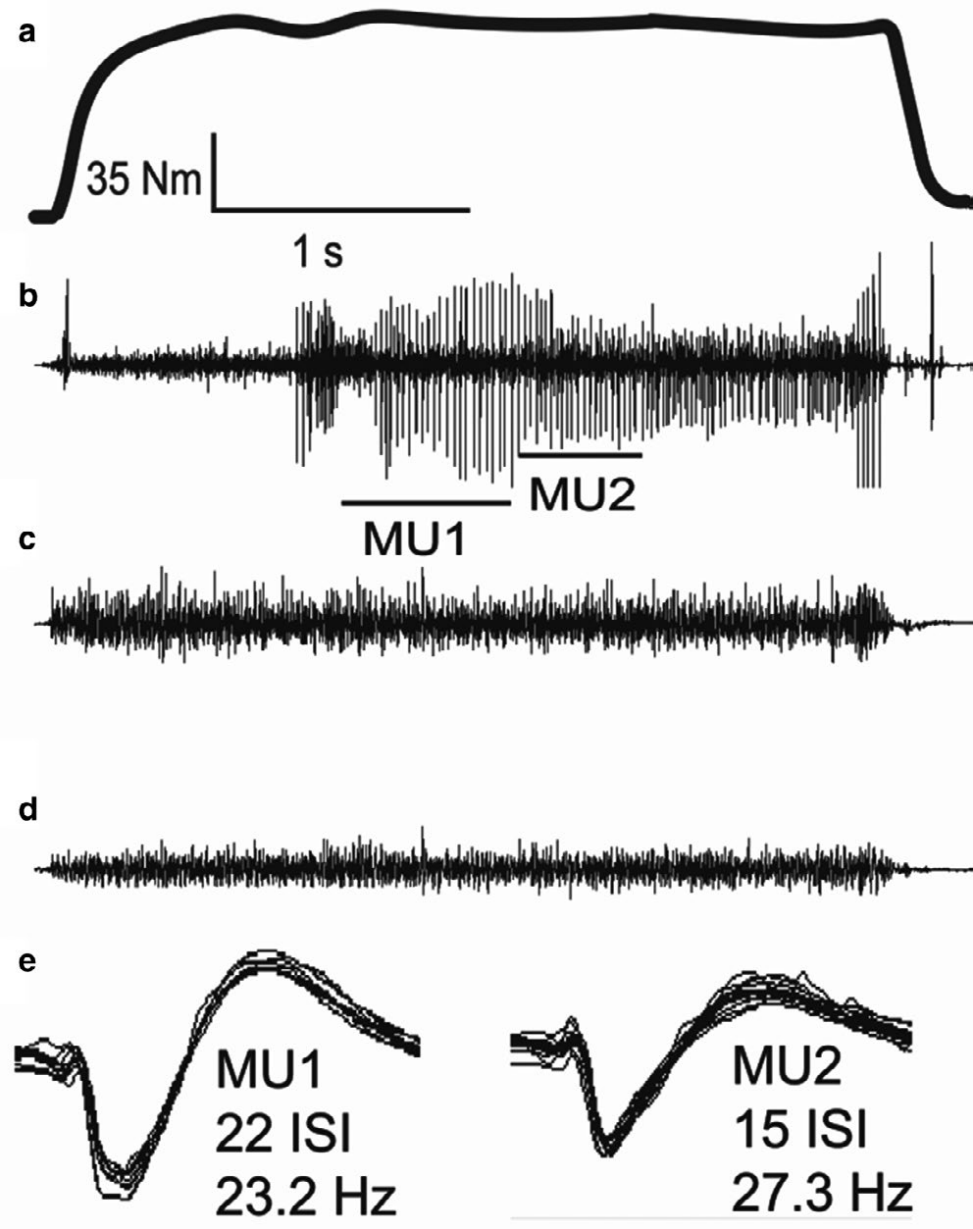
Example of motor unit (MU) action potential trains recorded at 100% maximal voluntary contraction with the ankle joint in the plantarflexed position. (a) Voluntary isometric torque. (b) Intramuscular electromyography recorded using a tungsten intramuscular electrode into the medial gastrocnemius. (c) Surface electromyography recorded from the triceps surae. (d) Surface electromyography recorded from the tibialis anterior muscle for coactivation. (e) Overlay of motor unit 1 (MU1; 22 interspike intervals; motor unit discharge rate 23.2 Hz) and motor unit 2 (MU2; 15 interspike intervals; motor unit discharge rate 27.3 Hz) action potentials. Amplitude changes in unit recordings reflect purposeful small movements of the recording electrode to select different units during the contraction

The starting ankle joint position was randomly chosen prior to the initiation of the testing session. Participants performed two ~3 s isometric dorsiflexion MVCs to record maximal tibialis anterior activity for the coactivation sEMG normalization (details below). This was followed by two ~3 s isometric plantar flexion MVCs in order to establish the baseline maximal plantar flexion torque. If the difference between the first two dorsiflexion or plantar flexion MVC attempts was >5%, participants performed a third MVC. All maximal efforts were separated by at least 3 min to avoid fatigue. All participants were provided with strong verbal encouragement and visual feedback during the MVC attempts. In order to assess plantar flexion voluntary activation, a supramaximal electrical square pulse was delivered 1 s prior to, at the plateau region and 1 s following the plantar flexion MVC. Once baseline MVC values were determined, participants performed 3–10 s steady‐state contractions at four different contraction intensities (25%, 50%, 75%, and 100% MVC) in a pseudo‐randomized order with 30–180 s rest periods between contractions to avoid fatigue. The ankle joint was placed in a neutral position (0°) during the rest periods to avoid the effects of prolonged stretch or shortening on the muscles of the leg (Guissard et al., 1988; Trajano et al., 2014). Prior to the subsequent contraction, the ankle joint was returned to the testing position and the contraction was performed ~5 s after the change in ankle joint position. Motor units (MU) were sampled during the plateau region of the contractions (Figure 1). To ensure collection of as many discrete MU as possible, each microelectrode was manipulated and advanced slowly through the muscle during the contraction. Manipulation involved small changes in position with later lateral pressures on the needle shaft in addition to small advancements into the muscle at a rate of 1–3 mm per contraction (Bigland‐Ritchie et al., 1992). After a series of contractions, the microelectrodes were reinserted at a different location separately into each of the three muscles to decrease the possibility of recording from the same MU (Rich et al., 1998). During one session, MU were recorded from all three muscles during all contraction intensities. Several recordings were made at each contraction intensity until an MVC contraction was reduced to 95% of the baseline MVC, likely indicating fatigue and the session ended. This technique has been used extensively in the previous literature (Bigland‐Ritchie et al., 1992; Dalton et al., 2009; Kennedy & Cresswell, 2001; Kirk & Rice, 2017; Rich & Cafarelli, 2000) and has proved to be very successful during high‐intensity contractions to allow selective MU recordings. Actively moving the recording microelectrode during contractions allows for recording from many different MUs. Participants returned to the lab a minimum of four times in order to maximize the number of MU recorded from all three TS muscles creating an adequate profile of MUDRs of their MU pools.

### 2.5 Data acquisition and analyses

Analysis was performed offline using Spike2 (Cambridge Electronic Design). For contractile properties of the evoked plantar flexion twitch, the following measurements were made: peak twitch amplitude, twitch time‐to‐peak tension, half relaxation time (HRT), contraction duration, and peak rates of torque development and relaxation. Voluntary activation was calculated using the interpolated twitch technique shown to be valid and reliable (Behm et al., 1996) and calculated as previously described by Todd et al., 2004. To assess maximal tibialis anterior neuromuscular activation, sEMG root‐mean‐squared (RMS) amplitude was calculated for a 1 s epoch at the plateau phase of a dorsiflexion MVC. This value was used to normalize equivalent epochs of tibialis anterior RMS sEMG for all other plantar flexion contraction intensities in order to assess coactivation. Submaximal (25%, 50%, 75% MVC) TS sEMG RMS was calculated for a 1 s epoch at the plateau phase of the plantar flexion contractions and normalized to a 1 s epoch at the plateau phase of a plantar flexion MVC in the same ankle joint position.

A template shape algorithm facilitated the process for the MU analysis, but visual inspection by an experienced operator was required to confirm spike allocation to each specific MU train. Inclusion criteria for MU included: consistent shape as viewed in a sequential overlay of MU potentials, and a minimum of five contiguous action potentials per MU train with an interspike interval coefficient of variation equal or less than 30% (Fuglevand et al., 1993).

### 2.6 Statistical analysis

Analysis was performed in R (version 3.4.3). A paired two‐tailed *t*‐test was used to compare voluntary activation, MVC torque and twitch characteristics between the PF and DF ankle joint positions. The effect sizes of the joint position on twitch characteristics were assessed using a Cohen's *D* test. The sEMG data for three subjects were removed from the statistical analysis due to technical problems during data acquisition. A three‐way analysis of variance was used to compare the normalized sEMG for the TS and tibialis anterior (coactivation) across all plantar flexion contraction intensities between the two ankle joint positions. A Tukey post hoc significance test was used to assess where the differences in coactivation exist. These data are reported as mean ± standard deviation.

For MUDR comparisons, a mixed linear model was constructed using the lme4 package (Bates et al., 2015). In this model, we included MUDRs as the dependent variable with ankle joint position (DF and PF) and contraction intensity (25%, 50%, 75%, 100% MVC) as fixed effects. We accounted for the inter‐subject and day‐to‐day variability in MUDRs by including participants, MVC and day of testing as random effects. The statistical significance of the fixed effects (ankle joint position and contraction intensity) was tested by fitting the model with restricted maximum likelihood and deriving degrees of freedom via Satterthwaite approximation using the lmerTest package (Kuznetsova et al., 2017). When significance was found, we contrasted the estimated marginal means of the levels of significant effects with Tukey adjustments for multiple comparisons using the emmeans package (updated version of lsmeans in Lenth, 2016). The MUDRs recorded from each muscle (MG, LG, and soleus) were analyzed separately. These data are reported in the text and displayed as least square means (95% confidence intervals). Alpha was set at 0.05.

## 3 RESULTS

### 3.1 Strength, voluntary activation, and contractile properties

Despite no difference in voluntary activation between the DF and PF ankle joint positions (*p* = 0.33; *D* = 0.2), plantar flexion MVC torque was 61% lower in the PF compared with the DF position (*p* < 0.001; *D* = 3.4). Similarly, peak twitch torque was 70% lower in the PF compared to the DF position (*p* < 0.001; *D* = 2.9). HRT was ~37% slower in the DF compared with the PF ankle joint positions (*p* < 0.001; *D* = 4.9), whereas time‐to‐peak torque (TPT) was ~5% faster in the PF compared with the DF position (*p* = 0.16; *D* = 0.5). Thus, overall contraction duration (TPT + HRT) was significantly lower in the PF compared with the DF position (*p* < 0.001; *D* = 3.7). Normalized maximal rate of torque development and maximal rate of torque relaxation (s^−1^) were calculated by dividing the peak rate of torque development and peak rate of torque relaxation (Nm/s) by the twitch peak torque (Nm), respectively. Normalized maximal rate of torque development was not statistically different between the two positions (*p* = 0.34; *D* = 0.4), whereas normalized maximal rate of torque relaxation was significantly faster in the PF compared to the DF position (*p* < 0.001; *D* = 4.9; Table 1).

**TABLE 1:** Mean plantar flexion maximal voluntary contraction (MVC) torque, voluntary activation (VA), and twitch contractile properties (CD, contraction duration (TPT + HRT); HRT, half relaxation time; NMRR, normalized maximal rate of torque relaxation; NMRTD, normalized maximal rate of torque development; Pt, peak twitch torque; TPT, time‐to‐peak torque) at two different ankle joint positions. PF refers to a plantar flexed ankle joint at 20°. DF refers to a dorsiflexed ankle joint at 20°

Parameter	DF	PF	Effect size (*D*)
MVC (Nm)	284.8 ± 65	112.7 ± 31.1*	3.4
VA (%)	94.5 ± 5.1	95.6 ± 6.4	0.2
Pt (Nm)	39.5 ± 12.5	11.9 ± 4.8*	2.9
TPT (ms)	109.2 ± 10.7	103.5 ± 11.0	0.5
HRT (ms)	100.9 ± 7.2	64.2 ± 7.7*	4.9
CD (ms)	210.1 ± 12.5	167.7 ± 10.3*	3.7
NMRTD (s^−1^)	16.2 ± 1.0	16.9 ± 2.1	0.4
NMRR (s^−1^)	−12.2 ± 1.3	−7.5 ± 0.4*	4.9

Note

Values are reported as mean ± standard deviation. Effect sizes are calculated using a Cohen's *D* test.

* Signifies the value is significantly different between positions (*p* < 0.05).

### 3.2 Electromyography

A significant interaction was detected between muscle × contraction intensity × joint position for the normalized sEMG values recorded in only seven subjects. No differences were detected for TS normalized sEMG at 25 (*p* = 0.42), 50 (*p* = 0.08), and 75% MVC (*p* = 0.54) between the two ankle joint positions. Tibialis anterior coactivation normalized sEMG showed no significant differences at 25 (*p* = 0.91) and 50 (*p* = 0.15) % MVC plantar flexion contractions in the DF (9.2 ± 4.0% and 16.6.0 ± 6.7%, respectively) compared with the PF (14.0 ± 4.7% and 26.9 ± 8.8%, respectively) position, however, it was significantly lower at 75 (*p* < 0.001) and 100 (*p* < 0.001) % MVC plantar flexion contractions in the DF (25.4 ± 9.3% and 38.4 ± 13.6%, respectively) compared with the PF (42.7 ± 11.9% and 68.1 ± 16.2%, respectively) ankle joint position.

**FIGURE 2:**
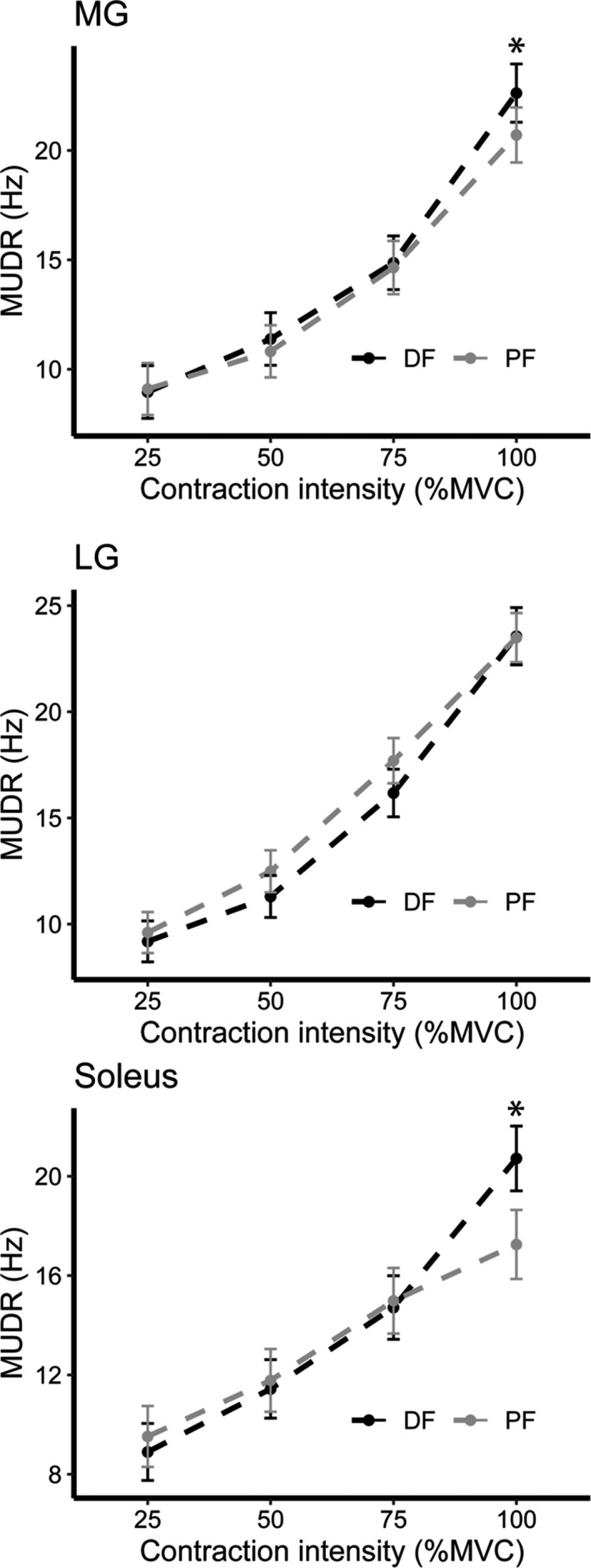
Motor unit discharge rates from the medial gastrocnemius (MG), lateral gastrocnemius (LG), and soleus across two ankle joint positions for each contraction intensity set based on plantar flexed maximal voluntary contraction (MVC) torque. PF refers to a plantarflexed ankle joint at 20°. DF refers to a dorsiflexed ankle joint at 20°. Data are reported as means (95% confidence interval). *Signifies the value is significantly different between positions (*p* < 0.05)

A total of 2273 MU were recorded from the three muscles of the TS combined including both ankle joint positions (1144 in DF and 1129 in PF). The number of MG, LG, and soleus MU identified from each muscle across all four contraction intensities is given in Table 2. The MUDRs mixed linear models identified a significant interaction between contraction intensity and ankle joint position in the MG (*p* = 0.007) and soleus (*p* < 0.001), but not in the LG (*p* = 0.21). In the MG, MUDRs showed no differences between the ankle joint positions at 25 (*p* = 0.74), 50 (*p* = 0.12), and 75% MVC (*p* = 0.64), but at 100% MVC were ~9% higher in the DF compared with the PF position (*p* = 0.001). In the soleus, MUDRs showed no significant differences between the ankle joint positions at 25 (*p* = 0.36), 50 (*p* = 0.63), and 75% MVC (*p* = 0.73), but at 100% MVC were ~20% higher in the DF compared with the PF position (*p* < 0.001). All three muscles showed a significant increase in MUDRs with contraction intensity (*p* < 0.001) in both ankle joint positions (Figure 2).

**TABLE 2:** Parameters of motor unit trains identified from the medial gastrocnemius (MG), lateral gastrocnemius (LG), and soleus at the two ankle joint positions for each contraction intensity binned as a percentage of the maximal voluntary contraction (MVC) plantar flexion torque. PF refers to a plantarflexed ankle joint at 20°. DF refers to a dorsiflexed ankle joint at 20°. # of MU refers to the total number of motor units collected. MU/person refers to the mean number of motor units identified from each individual. # of ISI refers to the mean number of interspike intervals used to assess the discharge rate of a motor unit train. CoV refers to the coefficient of variation in the interspike intervals used to assess the discharge rate of a motor unit train. MVC% refers to the plateau torque at which the motor units were collected

Parameter	Muscle	Contraction intensity bins							
		25% MVC		50% MVC		75% MVC		100% MVC	
		DF	PF	DF	PF	DF	PF	DF	PF
# of MU	MG	149	129	129	118	91	85	44	63
MU/person		6.1 ± 2.8	5.6 ± 2.5	5.5 ± 2.9	5.7 ± 2.8	5.1 ± 2.7	6.2 ± 2.7	6.5 ± 2.3	6.1 ± 2.8
# of ISI		8.2 ± 4.6	8.2 ± 4.7	7.2 ± 4.4	7.5 ± 4.1	6.9 ± 4.2	6.7 ± 3	6 ± 1.9	6.3 ± 4
CoV		9.3 ± 4.2	10.2 ± 4.6	10.2 ± 5	10.8 ± 4.8	10.7 ± 4.8	12.3 ± 5.1	13.8 ± 5.5	14 ± 5.8
MVC%		23.4 ± 2.6	24.0 ± 1.9	47.7 ± 2.9	47.7 ± 2.2	72.9 ± 3.8	72.7 ± 3.4	97.6 ± 2.8	98.4 ± 4.5
# of MU	LG	134	118	89	96	51	60	28	43
MU/person		5.8 ± 2.7	6.1 ± 2.7	5.9 ± 2.6	6.4 ± 3.1	5.9 ± 2.8	6.0 ± 2.7	6.6 ± 2.7	6.2 ± 2.9
# of ISI		8.6 ± 5.1	7.8 ± 5.2	7.5 ± 4.6	8.8 ± 5.2	5.9 ± 2.4	6.3 ± 3	5.8 ± 2.2	6.3 ± 3
CoV		10.3 ± 5.8	10.1 ± 5	11.7 ± 5.8	12.1 ± 4.3	11 ± 5.4	13.1 ± 5.6	12.1 ± 4.9	13.3 ± 6.3
MVC%		24.1 ± 1.8	24.2 ± 1.9	48.6 ± 1.5	48.3 ± 2.1	72.3. ±3.1	73.8 ± 3.1	97.5 ± 3.2	99.5 ± 5.4
# of MU	Soleus	191	178	126	106	58	64	54	45
MU/person		5.8 ± 2.9	6.4 ± 2.6	5.6 + 3.3	6.3 ± 2.8	6 + 2.9	6.1 ± 2.6	5.3 ± 2.9	6.3 ± 2.4
# of ISI		7 ± 3.3	7.9 ± 4.9	7.4 ± 5.3	7 ± 3.5	7.1 ± 6	6.4 ± 3	5.7 ± 3.0	6.6 ± 3
CoV		8.6 ± 4.1	9 ± 4.7	9.7 ± 5.1	10.2 ± 4.6	10.2 ± 4.7	11.8 ± 5.2	12.1 ± 5	11.8 ± 5.7
MVC%		24.2 ± 3.3	23.9 ± 2.1	48.3 ± 3.2	47.8 ± 2.1	72.0 ± 3.9	73.1 ± 3.5	97.1 ± 3.3	98.7 ± 5.0

Note

Values are reported as mean ± standard deviation.

## 4 DISCUSSION

The purpose of this study was to compare the neuromuscular properties of the three components of the TS between two ankle joint positions: 20° PF (shortened TS muscles) and 20° DF (lengthened TS muscles). Plantar flexion maximal voluntary torque was 61% lower in the PF position, despite no changes in voluntary activation of the plantar flexors between the two ankle joint positions. Additionally, peak twitch torque was lower and HRT, contraction duration and normalized maximal rate of torque relaxation were faster in the PF position. Normalized sEMG showed significantly higher tibialis anterior coactivation during plantar flexion contractions in the PF compared with the DF position, whereas no changes were detected in TS normalized sEMG between the two ankle joint positions. Finally, MG and soleus MUDRs were higher in the DF compared with the PF position only at 100% MVC, whereas there were no differences in LG MUDRs between the two ankle joint positions. In all three muscles, mean MUDRs increased with contraction intensity.

Previous reports on the voluntary activation of plantar flexors (Kluka et al., 2016), abductor digiti minimi, elbow flexors, and tibialis anterior (Gandevia & Mckenzie, 1988) across different muscle lengths have also reported high voluntary activation levels independent of muscle length, but with a lower maximal torque in the shortened position. Peak twitch torque was also lower when the TS muscles were shortened as has been previously reported in the literature (Kluka et al., 2016; Landin et al., 2015; Sale et al., 1982). Our findings provide further evidence that the central nervous system is capable of fully activating the ankle plantar flexors maximally regardless of ankle joint position within the limitations of the interpolated twitch technique (Todd et al., 2004). The differences in strength between the two positions can be explained by the suboptimal overlap of actin and myosin due to the shortening of the TS components in the PF compared with the DF position, as indicated by the cross‐bridge theory of contraction (Huxley, 1957). In this study, we did not make direct measurements of muscle fiber lengths, but have assumed muscle length changes due to alterations of the ankle joint position. In support of this assumption, previous work exploring muscle length changes has reported shorter MG, LG, and soleus muscle fiber lengths when the ankle joint is placed in a PF compared with a DF position with the knee joint flexed at 90° (Kawakami et al., 1998). In addition to a decrease in twitch torque, plantar flexion contractile properties were faster when the muscles were in the shortened (PF) position. Similar findings have been previously reported in single isolated frog muscle fibers (Edman & Flitney, 1982), isolated cat muscle (Rack & Westbury, 1969) and in human muscles such as the tibialis anterior (Bigland‐Ritchie et al., 1992), abductor digiti minimi, elbox flexors (Gandevia & Mckenzie, 1988), hamstrings (Kirk & Rice, 2017), and ankle plantar flexors (Marsh et al., 1981; Sale et al., 1982). The slowing of supramaximal twitch contractile properties and higher electrical stimulation rates required to reach tetanic torque fusion at shorter muscle lengths (Gandevia & McKenzie, 1988; Kirk & Rice, 2017; Marsh et al., 1981) suggest that higher maximal MUDRs may be required to generate a MVC at a shorter muscle length.

In agreement with other studies (Bigland‐Ritchie et al., 1992; Del Valle & Thomas, 2004; Kirk & Rice, 2017), mean MUDRs were higher at greater levels of contraction intensity in the MG, LG, and soleus during plantar flexion contractions at both ankle joint positions (Figure 2). Therefore, rate coding appears to be a contributing mechanism throughout the range of isometric contractile intensity. For contraction intensities up to 75% MVC, no differences in MUDRs were detected between the two joint positions. At 100% MVC, MUDRs in the MG and soleus were greater in the DF position. Previous work reports either no differences in maximal MUDRs between the lengthened and shortened tibialis anterior (Bigland‐Ritchie et al., 1992) or higher maximal MUDRs in the shortened compared with the lengthened hamstrings (Kirk & Rice, 2017). In these studies, the muscles tested remained in the stretched or shortened position throughout the entire experimental procedure. It has been previously reported that static stretch of the muscle causes an inhibition of the motor neuron pool (Guissard et al., 1988) and a decrease in maximal neural drive (Trajano et al., 2014). As such, recorded MUDRs could have been inhibited due to the prolonged stretch demonstrating no change (Bigland‐Ritchie et al., 1992) or a decrease (Kirk & Rice, 2017) in the lengthened compared with the shortened muscle. To mitigate the effects of prolonged passive stretching of the TS muscles in this study, the ankle joint was placed in a neutral position (0°) during the rest periods between subsequent contractions. The higher MG and soleus maximal MUDRs in the lengthened position can likely be explained by the increased Ia afferent feedback to the MG and soleus motor neuron pool due to the stretch placed on the muscle fibers in the lengthened position.

Furthermore, it has been previously reported that the persistent inward current amplitude, which serves to amplify the synaptic input received at the motor neuron dendrites, was higher when the ankle was in a flexed compared with an extended position in the TS motor neurons of the adult cat (Hyngstrom et al., 2007). When the antagonist (tibialis anterior and extensor digitorum longus) tendons were cut, the persistent inward current amplitude showed no difference between the different ankle joint positions. This provides evidence that Ia reciprocal inhibition from the antagonist muscle group has a fundamental role in modulating persistent inward current, thus affecting motor neuron excitability across different ankle joint positions (Hyngstrom et al., 2007). In accordance, our data show that tibialis anterior coactivation was higher during plantar flexion contractions in the PF position, which leads to increased Ia inhibitory input from the antagonist to the TS motor neuron pool, thus decreasing MUDRs in the shortened TS muscles. Although coactivation in leg muscles during isometric contractions can be affected by cross‐talk from agonist muscles (Raiteri et al., 2015), the TS sEMG in this study was not different between the two ankle joint positions and tibialis anterior sEMG was higher in the PF compared with the DF position in all subjects at contraction intensities of 75% and 100% MVC. Thus, differences in tibialis anterior sEMG are likely due to increased coactivation in the PF position as opposed to differences in agonist activation. The increased reciprocal inhibition may serve an important role in modulating the motor neuron excitability of the TS during the swing phase of gait, which begins with the ankle in the PF position, where these muscles are shortened.

It is worth noting that there is a wide range of afferent feedback affecting a motor neuron pool. Muscle spindles are composed of neurons that are sensitive to both dynamic and static stretch, with the latter exerting an effect on both the primary (type Ia) and secondary (type II) afferents (Matthews, 1962). Muscle tension is detected through Golgi tendon organ feedback (Houk & Henneman, 1967) and thus that input is likely different at the two muscle lengths given the differences in force production between the ankle joint positions. This study is unable to differentiate between the various possible sources of afferent feedback regulating the changes in MUDRs between the two ankle joint positions. Thus, it is reasonable to assume that these changes are a result of an interplay of all afferent feedback sources affecting the TS motor neuron pool.

Surprisingly, we found no differences in LG maximal MUDRs between the DF and PF positions. Previous work has shown that the LG demonstrates higher motor unit recruitment thresholds compared with the MG and soleus when participants perform voluntary plantar flexion contractions (Hali et al., 2020; Héroux et al., 2014), suggesting that the LG is comprised of higher threshold MU compared to the other muscles of the TS. An inverse relationship exists between Ia afferent feedback and the size of a motor neuron, meaning that lower threshold, small type motor neurons receive stronger Ia feedback compared with higher threshold, larger motor neurons (Windhorst & Kokkoroyiannis, 1991). Given the lack of a muscle length effect on LG MUDRs, our findings provide further support for the speculation that higher threshold motor neurons innervate this muscle.

There are many discrepancies in the literature regarding the effect of muscle length on MUDRs as noted in the introduction. The reasons for these discrepancies remain unclear, however they can include the different EMG recording techniques used, the relative amount of shortening experienced by each muscle and task specificity likely varies among the different muscles. As such, a study recording MUDRs during contractions at different muscle lengths in multiple muscles from the same individuals, while controlling for the effect of prolonged muscle stretch on the motor neuron pool (Guissard et al., 1988; Trajano et al., 2014), may be necessary to gain a better understanding of how muscle length affects MUDRs. Additionally, sex‐based differences in tendon compliance (Intziegianni et al., 2017) suggest there may be differences in how muscle length is altered with ankle joint position in males and females, thus leading to an altered neural response.

This study compared the effect of the ankle joint position on the neuromuscular properties of the TS muscle group. Consistent with previous results, we report decreased strength, twitch torque, and faster contractile properties when the TS muscles were shortened. Prior studies manipulated length changes in this muscle group by knee joint changes, thus not assessing the effect on soleus and furthermore did not record maximal MUDR changes with alterations in muscle length. Additionally, our results demonstrate that when eliminating the effect of prolonged static stretch, maximal MUDRs are higher in the MG and soleus at a lengthened compared with a shortened muscle length. It is possible this is a result of the increased Ia afferent input to the muscle motor neuron pool and decreased inhibitory inputs from the antagonist muscles at the lengthened position. Lastly, our findings indicate that LG MUDRs are similar at both ankle joint positions, providing further support for a differential activation between gastrocnemii heads as observed in other studies in different tasks.

## CONFLICT OF INTEREST

The authors declare no conflicts of interest.

## AUTHORS’ CONTRIBUTIONS

All authors contributed to the planning of this study. K.H. and A.M.Z. were involved in data collection. K.H. and A.M.Z. performed the data analysis. K.H. prepared and drafted the paper. All authors contributed to and approved the final draft.

## References

[phy214680-bib-0001] Banks, R. W. (2006). An allometric analysis of the number of muscle spindles in mammalian skeletal muscles. Journal of Anatomy, 208, 753–768. 16761976 10.1111/j.1469-7580.2006.00558.x PMC2100235

[phy214680-bib-0002] Bates, D. M. , Bolker, B. , & Walker, S. (2015). Fitting Linear Mixed‐Effects Models Using lme4. ) Journal of Statistical Software, 67(1), 1–48. 10.18637/jss.v067.i01

[phy214680-bib-0003] Behm, D. G. , St‐Pierre, D. M. M. , & Perez, D. (1996). Muscle inactivation: An assessment of the interpolated twitch technique. Journal of Applied Physiology, 81(5), 2267–2273. 8941554 10.1152/jappl.1996.81.5.2267

[phy214680-bib-0004] Bigland‐Ritchie, B. , Furbush, F. H. , Gandevia, S. C. , & Thomas, C. K. (1992). Voluntary discharge frequencies of human motoneurons at different muscle lengths. Muscle and Nerve, 15(2), 130–137. 1549135 10.1002/mus.880150203

[phy214680-bib-0005] Christova, P. , Kossev, A. , & Radicheva, N. (1998). Discharge rate of selected motor units in human biceps brachii at different muscle lengths. Journal of Electromyography & Kinesiology, 8(5), 287–294. 9785249 10.1016/s1050-6411(97)00034-5

[phy214680-bib-0006] Dalton, B. H. , Harwood, B. , Davidson, A. W. , & Rice, C. L. (2009). Triceps surae contractile properties and firing rates in the soleus of young and old men. Journal of Applied Physiology, 107, 1781–1788. 19797692 10.1152/japplphysiol.00464.2009

[phy214680-bib-0007] Del Valle, A. , & Thomas, C. K. (2004). Motor unit firing rates during isometric voluntary contractions performed at different muscle lengths. Canadian Journal of Physiology and Pharmacology, 82(8–9), 769–776. 15523534 10.1139/y04-084

[phy214680-bib-0008] Edman, K. A. P. , & Flitney, F. W. (1982). Laser diffraction studies of sarcomere dynamics during “isometric” relaxation in isolated muscle fibres of the frog. Physiol (Lord), 329, 1–20. 10.1113/jphysiol.1982.sp014287 PMC1224764 6982971

[phy214680-bib-0009] Fuglevand, A. J. , Winter, D. A. , & Patla, A. E. (1993). Models of recruitment and rate coding organization in motor‐unit pools. Journal of Neurophysiology, 70(6), 2470–2488. 8120594 10.1152/jn.1993.70.6.2470

[phy214680-bib-0010] Gandevia, S. C. , & Mckenzie, D. K. (1988). Activation of human muscles at short muscle lengths during maximal static efforts. Journal of Physiology, 407(1), 599–613. 10.1113/jphysiol.1988.sp017434 PMC1191222 3256627

[phy214680-bib-0012] Guissard, N. , Duchateau, J. , & Hainaut, K. (1988). Muscle stretching and motoneuron excitability. European Journal of Applied Physiology and Occupational Physiology, 58(1–2), 47–52. 3203674 10.1007/BF00636602

[phy214680-bib-0013] Hali, K. , Dalton, B. H. , Harwood, B. , Fessler, A. F. , Power, G. A. , & Rice, C. L. (2020). Differential modulation of motor unit properties from the separate components of the triceps surae in humans. Neuroscience, 428, 192–198. 31917353 10.1016/j.neuroscience.2019.12.023

[phy214680-bib-0014] Hali, K. , Kirk, E. A. , & Rice, C. L. (2019). Effect of knee joint position on triceps surae motor unit recruitment and firing rates. Experimental Brain Research, 237(9), 2345–2352. 31292695 10.1007/s00221-019-05570-7

[phy214680-bib-0015] Héroux, M. E. , Dakin, C. J. , Luu, B. L. , Iglis, J. T. , & Blouin, J. S. (2014). Absence of lateral gastrocnemius activity and differential motor unit behavior in soleus and medial gastrocnemius during standing balance. Journal of Applied Physiology, 116, 140–148. 24311748 10.1152/japplphysiol.00906.2013 PMC3921363

[phy214680-bib-0016] Houk, J. , & Henneman, E. (1967). Responses of Golgi tendon organs to active contractions of the soleus muscle of the cat. Journal of Neurophysiology, 30, 466–481. 6037588 10.1152/jn.1967.30.3.466

[phy214680-bib-0017] Huxley, A. F. (1957). Muscle structure and theories of contraction. Progress in Biophysics and Biophysical Chemistry, 7, 255–318. 13485191

[phy214680-bib-0018] Hyngstrom, A. S. , Johnson, M. D. , Miller, J. F. , & Heckman, C. J. (2007). Intrinsic electrical properties of spinal motoneurons vary with joint angle. Nature Neuroscience, 10(3), 363–369. 17293858 10.1038/nn1852

[phy214680-bib-0019] Intziegianni, K. , Cassel, M. , Hain, G. , & Mayer, F. (2017). Gender differences of Achilles tendon cross‐sectional area during loading. Sports Medicine International Open, 1(4), E135–E140. 10.1055/s-0043-113814 30539098 PMC6226073

[phy214680-bib-0020] Johnson, M. A. , Polgar, J. , Weightman, D. , & Appelton, D. (1973). Data on the distribution of fibre types in thirty‐six human muscles. Journal of the Neurological Sciences, 18, 111–129. 4120482 10.1016/0022-510x(73)90023-3

[phy214680-bib-0021] Kawakami, Y. , Ichinose, Y. , & Fukunaga, T. (1998). Architectural and functional features of human triceps surae muscles during contraction. Journal of Applied Physiology, 85(2), 398–404. 9688711 10.1152/jappl.1998.85.2.398

[phy214680-bib-0022] Kennedy, P. M. , & Cresswell, A. G. (2001). The effect of muscle length on motor unit recruitment during isometric plantar flexion in humans. Experimental Brain Research, 136(4), 58–64. 10.1007/s002210000623 11310172

[phy214680-bib-0023] Kirk, E. A. , & Rice, C. L. (2017). Contractile function and motor unit firing rates of the human hamstrings. Journal of Neurophysiology, 117(1), 243–250. 27784806 10.1152/jn.00620.2016 PMC5220116

[phy214680-bib-0024] Kluka, V. , Martin, V. , Vicencio, S. G. , Giustiniani, M. , Morel, C. , Morio, C. , Ratel, S. (2016) Effect of muscle length on voluntary activation of the plantar flexors in boys and men. European Journal of Applied Physiology, 116(5), 1043–1051. 27032806 10.1007/s00421-016-3362-6

[phy214680-bib-0025] Kuznetsova, A. , Brockhoff, P. B. , & Christensen, R. H. B. (2017). lmerTest package: Tests in linear mixed effects models. Journal of Statistical Software, 82(13), 1–26.

[phy214680-bib-0026] Landin, D. , Thompson, M. , & Reid, M. (2015). Knee and akle joint angles influence the plantarflexion torque of the gastrocnemius. Journal of Clinical Medicine and Research, 7(8), 602–606. 10.14740/jocmr2107w PMC4471746 26124905

[phy214680-bib-0027] Lauber, B. , Lichtwark, G. A. , & Cresswell, A. G. (2014). Reciprocal activation of gastrocnemius and soleus motor units is associated with fascicle length change during knee flexion. Physiological Reports, 2(6), 1–10. 10.14814/phy2.12044 PMC4208651 24920126

[phy214680-bib-0028] Lenth, R. V. (2016). Least‐squares means: the R package lsmeans. Journal of Statistical Software, 69(1), 1–33.

[phy214680-bib-0029] Linden, D. W. V. , Kukulka, C. G. , & Soderberg, G. L. (1991). The effect of muscle length on motor unit discharge characteristics in human tibialis anterior muscle. Experimental Brain Research, 84(1), 210–218. 1855559 10.1007/BF00231776

[phy214680-bib-0030] Marsh, E. , Sale, D. , McComas, A. J. , & Quinlan, J. (1981). Influence of joint position on ankle dorsiflexion in humans. Journal of Applied Physiology, 51(1), 160–167. 7263411 10.1152/jappl.1981.51.1.160

[phy214680-bib-0031] Matthews, P. B. (1962). The differentiation of two types of fusimotor fibre by their effects on the dynamic response of muscle spindle primary endings. Quarterly Journal of Experimental Physiology and Cognate Medical Sciences, 47, 324–333. 13933877 10.1113/expphysiol.1962.sp001616

[phy214680-bib-0033] Pasquet, B. , Carpentier, A. , & Duchateau, J. (2005). Change in muscle fascicle length influences the recruitment and discharge rate of motor units during isometric contractions. Journal of Neurophysiology, 94(5), 3126–3133. 16014788 10.1152/jn.00537.2005

[phy214680-bib-0035] Rack, P. M. H. , & Westbury, D. R. (1969). The effects of length and stimulus rate on tension in the isometric cat soleus muscle. Journal of Physiology, 204(2), 443–460. 10.1113/jphysiol.1969.sp008923 PMC1351563 5824646

[phy214680-bib-0036] Raiteri, B. J. , Cresswell, A. G. , & Lichtwark, G. A. (2015). Ultrasound reveals negligible cocontraction during isometric plantar flexion and dorsiflexion despite the presence of antagonist electromyographic activity. Journal of Applied Physiology, 118(10), 1193–1199. 25614599 10.1152/japplphysiol.00825.2014

[phy214680-bib-0037] Rich, C. , & Cafarelli, E. (2000). Submaximal motor unit firing rates after 8 weeks of isometric resistance training. Medicine and Science in Sports and Exercise, 32, 190–196. 10647548 10.1097/00005768-200001000-00028

[phy214680-bib-0038] Rich, C. , O′Brien, G. L. , & Cafarelli, E. (1998). Probabilities associated with counting average motor unit firing rates in active human muscle. Canadian Journal of Applied Physiology, 23(1), 87–94. 10.1139/h98-006 9494742

[phy214680-bib-0039] Sale, D. , Quinlan, J. , Marsh, E. , McComas, A. J. , & Belanger, A. Y. (1982). Influence of joint position on ankle plantarflexion in humans. Journal of Applied Physiology, 52(6), 1636–1642. 7107473 10.1152/jappl.1982.52.6.1636

[phy214680-bib-0040] Todd, G. , Gorman, R. B. , & Gandevia, S. C. (2004). Measurement and reproducibility of strength and voluntary activation of lower‐limb muscles. Muscle and Nerve, 29, 834–842. 15170616 10.1002/mus.20027

[phy214680-bib-0041] Trajano, G. S. , Seitz, L. B. , Nosaka, K. , & Blazevich, A. J. (2014). Can passive stretch inhibit motoneuron facilitation in the human plantar flexors? Journal of Applied Physiology, 117(12), 1486–1492. 25342705 10.1152/japplphysiol.00809.2014

[phy214680-bib-0042] Tucker, K. J. , & Türker, K. S. (2004). Muscle spindle feedback differs between the soleus and gastrocnemius in humans. Somatosensory and Motor Research, 21, 189–197. 15763904 10.1080/08990220400012489

[phy214680-bib-0043] Voss, H. (1971). Tabulation of the absolute and relative muscular spindle numbers in human skeletal musculature. Anatomischer Anzeiger, 129, 562–572. 4260484

[phy214680-bib-0044] Windhorst, U. , & Kokkoroyiannis, T. (1991) Interactions of recurrent inhibitory and muscle spindle afferent feedback during muscle fatigue. Neuroscience 43(1), 249–259. 1833667 10.1016/0306-4522(91)90432-n

[phy214680-bib-0045] Del Valle A. , Thomas C. K. (2004). Motor unit firing rates during isometric voluntary contractions performed at different muscle lengths. Canadian Journal of Physiology and Pharmacology, 82, (8‐9), 769–776. . 15523534 10.1139/y04-084

